# Hardware, Software, and Wetware Codesign Environment for Synthetic Biology

**DOI:** 10.34133/2022/9794510

**Published:** 2022-09-01

**Authors:** Samuel M. D. Oliveira, Douglas Densmore

**Affiliations:** ^1^Department of Electrical and Computer Engineering, Boston University, MA 02215, USA; ^2^Biological Design Center, Boston University, MA 02215, USA

## Abstract

Synthetic biology is the process of forward engineering living systems. These systems can be used to produce biobased materials, agriculture, medicine, and energy. One approach to designing these systems is to employ techniques from the design of embedded electronics. These techniques include abstraction, standards, modularity, automated design, and formal semantic models of computation. Together, these elements form the foundation of “biodesign automation,” where software, robotics, and microfluidic devices combine to create exciting biological systems of the future. This paper describes a “hardware, software, wetware” codesign vision where software tools can be made to act as “genetic compilers” that transform high-level specifications into engineered “genetic circuits” (wetware). This is followed by a process where automation equipment, well-defined experimental workflows, and microfluidic devices are explicitly designed to house, execute, and test these circuits (hardware). These systems can be used as either massively parallel experimental platforms or distributed bioremediation and biosensing devices. Next, scheduling and control algorithms (software) manage these systems’ actual execution and data analysis tasks. A distinguishing feature of this approach is how all three of these aspects (hardware, software, and wetware) may be derived from the same basic specification in parallel and generated to fulfill specific cost, performance, and structural requirements.

## 1. Introduction

Engineering frequently transforms a set of needs into a physical object that fulfills those needs. Crossing a river requires a bridge. Communicating around the world involves radio transmission. Traveling long distances requires aircraft. A crucial aspect in transforming needs into physical objects is a disciplined approach to the process so that it is safe, effective, and replicable. Needs must have requirements, and solutions must quantifiably meet those needs. As engineering fields mature, so do the engineering processes associated with them.

Bioengineering is maturing to a point where well-characterized genetic elements built from DNA can be artificially composed and introduced into a living organism, e.g., bacteria. This new DNA acts as an additional “program” that can harness the machinery in the bacteria [1] to make new fuels, therapeutics, materials, and biosensors [2]. Specifically, a field called “synthetic biology” emerged in the early 2000s, in which this process specifically leveraged abstraction, modularity, and standards. The central premise is that reusable “parts” could be combined to create “devices,” then composed into “systems.” In the process, the “design-build-test” cycle could be employed, allowing others to easily modify these designs if needed and for software and automation workflows to be introduced.

Some of the representative efforts in this space have been as follows: genetic circuits that implement Boolean logic [3], bacteria that enable agriculture advances [4], yeasts that produce biofuels [5], bacteria that build novel biomaterials [6], mammalian cells that produce therapeutics [7], microbial consortia that create synthetic biosensors [8], and innovative bioremediation solutions [9].

While developing these biological systems and their building blocks (wetware) focuses on experimental microbiology, a small community is concerned with developing software to support this process. These solutions can be associated with the specification, design, build, test, learn, and archive aspects of the workflow [10, 11]. “Biodesign automation” efforts have emerged where “biofoundries” attempt to provide modular services that begin to create an ecosystem where design is decoupled from a distributed fabrication network much in the same way that this is possible for semiconductor-based electronics. Software systems have also built genetic compilers [12], sophisticated modeling frameworks [13], DNA assembly planning software [10], and standardized data exchange formats [14].

Next, hardware is beginning to play a role in synthetic biology. This hardware includes laboratory automation equipment (e.g., liquid handling automation), test equipment (e.g., flow cytometry), and microfluidic systems [15]. Microfluidics are particularly interesting since they can be used to develop the design and act as an artificial environment in which highly specified biological systems can be freed from many of the highly specific environmental contexts of an actual deployment. For example, microfluidics have been used in high-throughput bioassays [16], DNA modular assembly [17], and portable devices for point-of-care applications [18].

**Figure 1:**
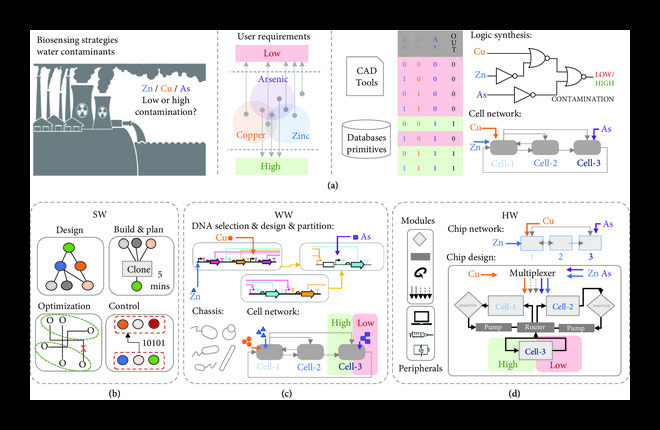
A single functional specification can be converted into software, wetware, and hardware components. (a) Using the example for detecting the presence of water contaminants, e.g., zinc (Zn), copper (Cu), and Arsenic (As), one can describe a potential abstract solution in terms of operation requirements (i.e., Venn diagram) and performance (i.e., truth table). Specialized tools help users interpret abstract descriptions into a series of instructions for designing, building, optimizing, testing, and screening biosensor candidates (i.e., cell networks) and microfluidic devices (i.e., chip designs) with the desired functionality. (b–d) We propose a “hardware, software, wetware” codesign ecosystem that guides the translation of user abstract descriptions into specific components for engineering novel wetware and hardware. (b) software “SW” tools aid in the design and implementation of wetware “WW” (e.g., DNA parts and devices) (c) and the design, fabrication, and control of hardware “HW” (e.g., microfluidic components and devices) (d). This is possible by employing techniques and principles from embedded electronics, including abstraction, standards, automated representation, transformation, and use of formalized semantic models of computation (e.g., Petri nets, process networks). In the end, characterized components and peripheral instruments are selected to perform the desired solution.

**Figure 2:**
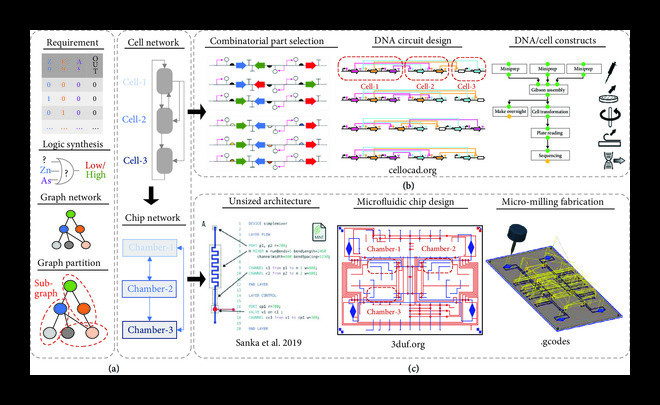
Software tools automate hardware and wetware engineering workflow from a single abstract description. (a) Many solutions for real-life problems can be described at different levels of abstraction, from graphical, high-level descriptions to possible logical relationships among variables and expected outputs (e.g., a truth table). Next, mathematical approaches and algorithms (i.e., graph network optimization and partitioning tools) can facilitate their conversion to physical implementations (i.e., potential DNA circuits and cellular systems, then the chip network and experimental requirements necessary for cell growth and signal exchange). (b) For a complete automation workflow of wetware creation, the first requirement is a genetic compiler capable of converting abstract descriptions into realizable DNA parts. Those parts are selected from libraries/databases of characterized DNA components, such as promoters and terminators. To physically realize DNA parts into devices, CAD tools (e.g., Cello 2.0) help users assign genetic parts and define their order following their expected functional roles. In addition, subgraphing tools can aid in the partitioning of large circuits into subgraphs. Finally, other tools help users define and select optimal assembly plans depending on the type/amount of DNA parts and DNA assembly methods. They can use the semantics of graph traversal to determine the type of laboratory protocol, equipment required, and other wetware (e.g., reagents, enzymes) to implement the DNA constructions (e.g., Aquarium). (c) Then, a combination of tools can help users specify, design, and fabricate microfluidic components into desired devices. They generally consider a library of characterized microfluidic components, roles, and constraints and recorded performance and precision to help specify the most appropriate designs and tests (image reused with permission from ref. Sanka, Crites, et al. 2019). Designs (e.g., multilayer microfluidic devices by the CAD tool 3DuF) are built following the list of components and dimensions previously and automatically defined, using fabrication instructions and specialized equipment, such as CNC micromilling machines.

This article outlines a vision where wetware, software, and hardware are thought about holistically in end-to-end design processes of particularly challenging biological problems. First, we present an outline for how, from a single, abstract description, a completely realized biological device with supporting hardware and software can be created much in the same way the embedded electronics are made with a “hardware, software, codesign” approach (Figure 1). Second, with the increasing complexity of biological problems, we present synthetic microbial communities as promising tools for synthetic biology applications due to their capability of labor division and spatial organization properties [19]. However, new design concepts and computational and genetic techniques must be developed to construct distributed functionalities in such cellular networks. Here, we present a series of sophisticated tools (Figure 2) that help users decompose complex biological tasks into the best combination of genetic circuits, cells, or communities and the performance-based microfluidic environments to help them achieve user-defined behaviors.

Finally, similar to the example illustrated in Figure 1, a recent study engineered an electrogenetic system of artificial microbial network of three bacterial species (a router, a verifier, and an actuator) and an external electrical device that can interrogate and control biological activity in real time [20]. If this approach becomes widely available, the world will see rapid progress in therapeutics, energy, materials, and biosensing challenges [21] across scales and biological contexts [19].

## 2. Background

The information to produce enzymes and proteins in all living organisms is stored in DNA molecules, transcribed into RNA, and then into proteins through translation reactions. These processes, along with other regulatory reactions [22], are at the heart of natural gene regulatory networks as they initiate, maintain, and regulate gene expression. Programmable genetic circuits are “engineered” biological networks that accept a set of inputs, often small molecules or proteins, and produce the desired output response [23]. For example, one class of genetic circuits is synthetic regulatory networks based on transcriptional control that generate output signals in response to sensed inputs (e.g., chemicals [24], light stimulus [25], and intercellular signals [26]. In addition, advances in synthetic biology tools [27] have allowed the forward engineering of such synthetic circuits in multiple living systems [28–30].

The construction of de novo functional DNA devices and their transformation into cells has been used to add external control over gene expression and cellular machinery [31]. Despite successful examples, such as digital logic gates [32], sequential logic circuits [33], and time-dependent dynamical circuits [34, 35] in bacterial cells, the rational development of synthetic DNA sequences is still challenging. First, most DNA construction frameworks are still time-consuming and error-prone. Second, although synthetic biologists have applied engineering principles to design DNA circuits using predictable frameworks [36, 37], synthetic engineering circuits still lack precise functional control when exposed to cell growth and varying intracellular contexts. In that, the availability of intracellular resources (e.g., ribosomes, RNA polymerase, and amino acids) required by synthetic constructs are shared with the host machinery (e.g., transcription, translation activity, cell density, and growth), which almost necessarily impacts DNA constructs’ functionality and predictability. Similarly, another possibility is that some synthetic constructs and regulators may affect the host growth and division. Finally, novel techniques have been developed to reduce the exogenous load genes have on the host and promote gene expression, e.g., a modified *Escherichia coli* strain with the selective allocation of mRNA degradation machinery [38]. See, e.g., the review [39] for more known implications and strategies to address them. Ideally, predictable DNA design tools must consider resource competition mechanisms and characterized variables as significant features in searching for intracellular context-dependent DNA design solutions.

Given the fact that biological processes have both functional requirements (what needs to be made) and structural constraints (how to realize it physically), one approach employs engineering principles and techniques from embedded electronics design [40]. First, the concepts of abstraction, standards, and modularity of well-characterized genetic components allow for functional genetic circuit “architectures,” including promoters, ribosome binding sites, and terminator sequences to be created. Second, the use of automated representation transformation and formal semantic models for such parts and corresponding assembled devices and systems allows for performance to be predicted [41, 42].

Embedded electronic system design is quite mature. For example, works now describe high-level design approaches where abstract functional requirements are translated into “models of computation.” These models include formally analyzable data structures like Petri nets, finite automata, and process networks [43]. Embedded system design environments include systems to create software and hardware from single descriptions and develop control software. In addition, it allows for system-on-a-chip (SoC) design environments at higher abstraction levels than RTL design environments like Verilog. These include SystemC [44], SystemVerilog [45], and SysML [46].

A codesign environment such as this would be very powerful if applied to synthetic biology. However, this environment would involve three elements instead of two, whereas in electronics, the platform is made of only semiconductors; in synthetic biology, this platform would include living and nonliving elements. Both elements would have unique design, build, and test workflow iterations but need to be designed in tandem (Figure 2). This presents an interdisciplinary opportunity that is likely to draw electrical and computer engineers into synthetic biology research in ways not previously possible and, in the process, help synthetic biology realize many of its early promises as the field looks for academic and commercial innovation.

## 3. Design Automation “Software” Workflow for Codeveloping “Hardware” and “Wetware”

We consider “software” tools as programming languages, computer scripts, databases, and software tools that enable (or improve) the wetware and the hardware engineering that meet user-defined descriptions (Figure 1). Automating how a series of formalized design transformations (best representing a particular problem) can have a potential solution ultimately implemented in biology and microfluidic fields is the process that is the paradigm shift we are advocating for. We believe that a set of “software” tools can help automate the generation of a consolidated user-defined solution that consists of “wetware” (i.e., DNA devices) and “hardware” (i.e., microfluidic devices) for a concrete problem (as, e.g., demonstrated in Figure 1(a)).

**Figure 3:**
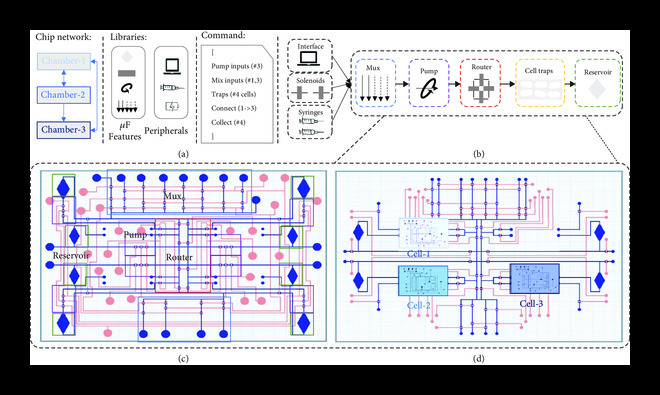
Complex microfluidic devices can be automatically designed and fabricated using modular components. (a) Given a chip network, software tools help users develop sophisticated microfluidic devices, from individual functionally characterized components, to enable independent cell culturing and cell-cell communication. Microfluidic very large-scale integrated (mVLSI) devices, for example, allow the continuous flow of liquids through microchannels and the stopping and routing of fluids by micromechanical valves, pumps, and multiplexers (b). A set of instruments (e.g., computers, syringes, and peristaltic pumps) can be selected for the implementing operations in a desired mVLSI device, i.e., activating/inactivating microfluidic valves. (c, d) In this hypothetical multilayer chip, top layers control the routing and placing of chemicals through flow paths and microvalves (by laser cutting or micromilling technologies). Then, a fourth independent layer is required for managing and maintaining cell growth (built by photolithography). Thus, another modular component, “cell traps” (d), can be designed and fabricated to act as a microenvironment for cells while communicating with liquid routed in mVLSIs.

**Figure 4:**
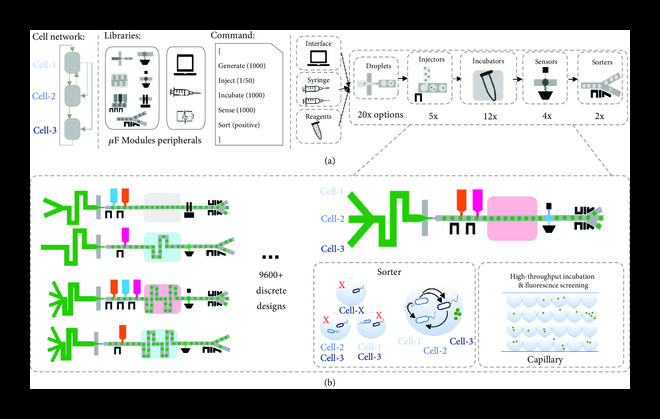
Droplet-based microfluidic devices can be automatically designed from low-cost components. (a) Given a cell network, droplet-based microfluidic devices can be built from individual components to implement their functionality. The performance of individual components is a fundamental step in designing such devices, and their characterization follows a thorough exploration of the design space and resulting performance. The process can be time-consuming and costly depending on the number of variables per component to be tested. (b) Low-cost, rapid prototyping methods enable a fast exploration of the design space. By varying component types, order, geometries, and distances, one can create numerous composed discrete designs with the same desired function but different and predictable performances. For the given cell network required, e.g., a high-throughput sorting, screening, and characterization device can be composed of cell injectors, incubators, and sorter components where the desired functions can be found.

For hardware, microfluidic components and complex devices associated with the user-defined descriptions are designed and fabricated using automated software workflows (Figure 2(b)). Here, following the case study presented in Figure 1, we present the software and constraints of continuous-flow multilayer mVLSI devices with spatial and temporal control for temporal dynamic studies of cells and communities (Figure 3) and, alternatively, how one can use a similar workflow to generate, sense, and sort droplets following bioassays’ requirements (Box 1 and Figure 4). The codesign example of Figure 1 illustrates the need for building a synthetic community of three bacterial species and developing a microenvironment that can promote individual cell growth and desired community behavior. Stable cocultivation systems should prevent faster-growing species from wiping out slower-growing ones. While standard methods (e.g., agar plates and liquid cultures) are straightforward, they cannot control the time, space, strength, and direction of cell growth and molecule exchange and increase the chance for interferences due to cell growth competition in dependence relationships (see, e.g., the review [47]).

**Box 1: Designing and building droplet-based microfluidic components and devices.**
Bioelectronic, droplet-based microfluidic devices hold promise to serve as high-throughput, parallelizable screening platforms for many applications in synthetic biology, such as selecting optimal microbiomes. For example, artificial environments, synthetic metabolic pathways [48], and synthetic communities can be developed and screened in search of specific metabolites, growth relations, or intercellular interactions.
Individual droplet microfluidic components have been developed to enable a wide range of functionalities, such as droplet generation [49], droplet sorting [50], droplet merging [51], droplet picoinjection [52], and droplet sensing [53] (Figure 4(a)). For example, by encapsulating standard biological chassis within monodisperse picoliter-scale emulsions, droplet microfluidic can grow, measure, and manipulate biological systems at the single-cell level with unprecedented throughput [54]. In addition, single cells can be encapsulated within droplets and incubated to divide a diverse library into monoclonal populations. This is a particular advantage for droplet microfluidics in synthetic community studies (Figure 4(b)). Despite its potential, the multidisciplinary nature of microfluidic platforms presents a significant technical challenge in designing, fabricating, and operating a functional device.
Once developed, each component needs to be characterized to map the design space to various performances. While this can be an experimentally expensive task, learning this behavior can be streamlined through computational modeling or orthogonal design of experiments (DoE) [55]. The latter is based on a design matrix that allows studying the relationship between multiple input variables to determine their effect on single or multiple responses. Alternatively, our group has used DoE [37], standard machine learning [56], and iterative active learning [57] to effectively explore the design space of flow-focusing droplet generators. Once the behavior is characterized, these robust components can be integrated into predictive software tools and scaled into complex microfluidic screening platforms.
Such devices combine microfluidic components, electronics, and optics in a software-driven environment to sense, learn from, and respond to complex biological signals. For example, similar systems have been shown to actively sense electrical signals (impedance, redox reactions, etc.) through integrated electrodes coupled to electrical sensors [58] or fluorescence detectors, use the measurement to refine in silico models, and subsequently provide feedback control through pneumatic or electrical actuators [59]. In addition, a high-voltage signal can be actively applied to electrodes to overcome the surface tension between two fluids and be used for controlled droplet coalescence [51] or picoinjection of a reagent reservoir into a passing droplet [60]; [61]. Next, a transient high-voltage signal can be applied to dielectrophoretically deflect passing droplets across streamlines to perform droplet sorting [52]. To fabricate such bioelectronic chips, novel methods have been developed using molten solder [62], liquid metal [63], saltwater [64], and conductive ink [65].

Spatial-temporally distributed chips (e.g., the multilayer mVLSI devices proposed here) are microfluidic systems with varying microfluidic chambers (i.e., monolayer traps for bacteria) that allow monoclonal and coculturing communities to grow and stay alive for short periods (e.g., hours or days) [66] while maintaining the exchange of chemicals and metabolites and preventing the effect of cell growth competition. Other microfluidic features, such as multilayer valves, can help keep cell types separate and route signals in a one-way communication manner (Figure 1, “HW” section). In the end, we believe that the proposed spatial-temporally distributed chips can be potentially applied to produce enzymes, fine chemicals, biopolymers, food additives, and antimicrobials, among others (see, e.g., the review [67]).

**Figure 5:**
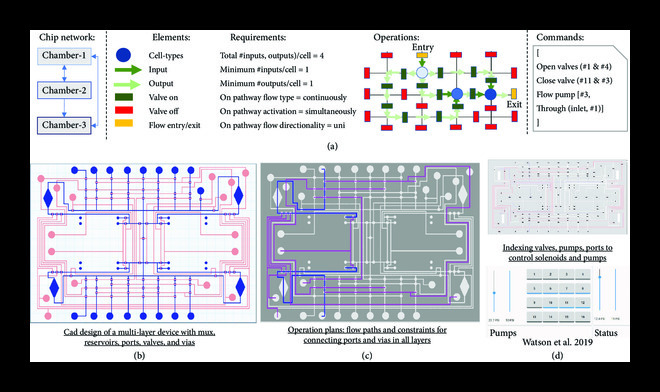
Software tools can plan and control multiple operational modes of complex microfluidic platforms. (a) Given a chip network and the requirements for cell(s), medium(a), and chemical(s), a graphical representation of the network can be determined. CAD tools for multilayer microfluidic devices help users define individual components and connections to create a complex layout (e.g., the combination of parallel liquid routers, valves, pumps, and ports). Complex flow and control operations can be simplified by mapping and connecting ports, inlets, and outlets in an abstract way (b). Other tools can design, plan (c), and control the desired operations in physical implementations (d) (Image reused with permission from https://hackaday.io/project/27511-microfluidics-control-system). Software design and control tools play an essential role in finding the best layout (control and flow pathways) and automatically creating the HW control scripts that enable the physical implementation of desired operations. Software tools can be tailored to control the functioning of mVLSI chips by assessing the number and location of valves, pumps, and ports. For a single or a collection of operation modes, the tool determines the chip’s state (on/off), then controls its functioning and dynamics over time through external peripheral controllers, e.g., pressure controllers and pumps.

Designing and controlling the operation of mVLSI devices are not trivial tasks. Software tools help determine the number and location of microvalves required by users for desired functions, e.g., the switch of fresh media temporal external perturbations, then define the set of pathways, valves, pumps, and ports that should be activated to perform such operations (Figure 5).

For wetware, genetic compilers transform user-defined descriptions into designed genetic circuits, thus helping researchers select optimal candidates for performing biological tasks. The automated construction of DNA sequences is achieved by genetic compilers that define DNA candidates following standardized data exchange formats (e.g., SynBioHub [14]). Next, optimized plans are generated by software tools (e.g., Puppeteer [10]) and implementation controlled by laboratory information management systems (e.g., Aquarium [68]) (Figure 2(c)).

### 3.1. Designing Sophisticated Microfluidic Devices from Characterized Components

Designing and fabricating hardware components and devices to perform biologically relevant tasks is not trivial. For example, technology-based software tools have assisted researchers in constructing microfluidic components based on engineering principles and design cycles using specific design and fabrication methods. For instance, similarly to hardware description languages used in electronics [69], researchers have developed microfluidic description languages [70] for the automated design of both control and flow layers from high-level descriptions [71]. However, they lack the flexibility to adopt multiple fabrication techniques from the same high-level abstract description of the solution [72]. “Fluigi” is an example software workflow that deals with this problem [73].

“Fluigi” allows the automated specification, design, and fabrication of microfluidic devices from a library of well-known characterized microfluidic components and high-level device specifications, i.e., user-defined descriptions of expected device operations and biological systems’ performance (Figure 2(b)). In the specific step, programming languages facilitate the interface between users and the entire workflow for those lacking programming skills and unfamiliar or engaged with design and fabrication techniques. A library of characterized microfluidic components, roles and constraints, and recorded performance and precision are considered by algorithms, models, and machine learning tools to help specify the most appropriate microfluidic devices for the next step. Future tools should refine and tune microfluidic components (“hardware”) based on their fluidic performance, considering the biological system’s desired output (“wetware”).

One of the “Fluigi” workflow tools in the design step is named 3DuF [74]. It is an open-source, interactive microfluidic system designer tool for integrating many components such as valves and pumps and building more complex microfluidic devices. In the build step, 3DuF allows for automatic generation of fabrication output files (in various formats), also obtained manually using commercial tools such as Fusion360 (Autodesk), required for the most relevant engineering techniques used for fast, low-cost fabrication of microfluidic chips, such as laser-cutters [75] and CNC milling microfluidic devices [76] and 2-layer thermoplastic [65].

This workflow has been extensively used for the rapid, low-cost, reproducible, and customized fabrication of complex multi-layer integration of spatial-temporal mVLSI devices [77] (as in Figures 2 and 3) and droplet-based thermoplastic microfluidic devices [56] (as in Figure 4).

For droplet-based microfluidic devices, a recently published open-source tool named DAFD was developed to predict microfluidic hardware features from droplet-based geometry and performance specifications [56]. Namely, the authors demonstrated how the refinement and tuning of component designs feed machine learning tools and training datasets (i.e., the relationship between component geometry and performance) to generate optimal strategies with predefined design performance rapidly.

For spatial-temporal mVLSI devices, algorithms have been developed to introduce computer-aided design tools for optimizing the design and control of spatially and temporally distributed microfluidic valves [18, 78]. Following specific timings, the programmatic combination of pathways and reagents makes biological interactions rationally. For example, a bottom-up engineering approach [79] with PR-PR software user-interface tool was used to build a microfluidic device that controls a reagent from a macro scale input well to output well through a selected pathway in the chip by activation of internal microscale valves.

Finally, as part of the performance assessment, biological systems can have their dynamical properties tracked and studied over time using time-lapse microscopy and signal processing tools. Microfluidic, microscopy set-ups can take deeper quantitative data on cellular behavior at a single level, thus improving the refinement of microfluidic component performance (especially those housing cells or fluorescently activated droplets) [80].

### 3.2. Designing and Engineering Large Genetic Constructs from Characterized Parts

We consider wetware as DNA parts, and their composed DNA devices are used to produce biological systems that meet user-defined abstract descriptions (Figure 1). Researchers have leveraged synthetic biology tools and methods [36] to develop novel DNA parts and devices to expand the possibilities of biological applications. Synthetic biology methods generally consider DNA parts as reusable building blocks of larger devices in assembly methods (see, e.g., the review [81]). In addition, researchers have made significant advances in other related fields, such as protein engineering (see, e.g., the review articles [82, 83]) and metabolic engineering (see, e.g., [84, 85]. In this article, due to the specific paradigm shift, we aim to demonstrate the most likely and achievable “wetware” to be predictably designed; thus, this section focuses on advances and challenges in designing, building, and testing DNA constructs from characterized DNA parts.

So, like electrical engineering principles, multiple sequence candidates must be studied and characterized with certain predictability and success rates before being used reliably and rationally in a genetic engineering process. Software tools, e.g., Cello version 1.0 [32], can serve as “genetic compilers” to transform user-defined high-level descriptions into engineered circuits with the expectation of performing predefined complex functions. Design tools that provide high-level descriptions to users have the promise to ease the adoption of intricate design and fabrication workflows by a wider user community. This is a fundamental characteristic of the paradigm shift we advocate for in this article (e.g., Figures 1 and 2). Yet, within this context, the development of open-source tools can contribute to creating a codesign environment of software design tools and expanding their features to reduce the adoption barrier to a broader range of users.

For example, the original Cello [32], launched in 2016, has been an important tool to demonstrate the vision of the codesign environment. However, in practice, it has limited protocols that users could use; e.g., the circuit design candidates could only be integrated into bacterial plasmids (with no possibility of genome integration). In addition, Cello v1 provided only a single curated library (i.e., User Constraint File (UCF)) of genetic components to work with designs implemented in a single bacteria host, *E. coli* (MG1655).

In 2022, Cello version 2.0 was launched with new features that could potentially increase its adoption by a wider community. To name a few features, Cello V2 provides expanded UCF libraries for up to five different host organisms. Then, if an organism is not found, the new UCF format allows users to design their own libraries. These characteristics increase Cello’s capabilities beyond bacterial plasmids to other organisms and the genome (Figure 2(c)). Finally, Cello 2.0 allows flexible descriptions of the logic gate structure and mathematical models expressing dynamic behavior, a new graphical user interface (easier to use for wider audiences), and a connection to the open-source software environment (SynBioHub) that works as a components’ repository. This integration demonstrates the importance of a codevelopment environment of software and wetware, ideally including hardware.

After a DNA circuit is designed, multiple DNA parts must be assembled and tested. Automated and software-oriented bioengineering frameworks [85] can speed up steps and increase the reliability of lab procedures (Figure 2(c)). The steps to design and build numerous combinations of DNA parts (before one finds a successful final construct) can be extensive, costly, and time-consuming. For example, high-order chromosomes (e.g., yeast and plants) can be constructed using DNA assembly methods. Twist Biosciences and GenScript have applied de novo synthesis to build multiple oligos effectively. However, *de novo* synthesis of large sequences, e.g., the yeast chromosome, is costly, time-consuming, complex to engineer, and problematic due to spurious depurination of synthesized oligomers [86, 87] that causes sequence polymorphisms.

To cope with costs, time, and sequence defects, researchers have attempted to design multiple DNA devices and transform them into live cells using molecular biology tools employed in an automated [88] instead of manual [89] method. In particular, automated approaches use standardized, scalable, modular cloning techniques for fast, high-throughput construction of multiple DNA devices and systems simultaneously from shared libraries of DNA parts [90]. Other computational tools use either in silico predictions of structural changes to design novel sequences, following physical constraints [91] or the *in vivo* dynamical properties of DNA devices and their performance limitations to define the best candidates [36, 92, 93].

To decrease the complexity of such large constructions, small-scale laboratories have opted for conventional cloning techniques or modular assembly methods combined with sequences designed using CAD tools. However, the wide use of multiple uncoordinated technologies has contributed to an increasing lack of formalized design processes, standardized metrics, terminology, protocols, and good practices. Combined, they have negatively impacted, for example, the reproducibility and replicability of scientific results, which highlights the importance of creating standardized protocols and sharing such detailed experimental data, thus determining robust construction pipelines.

Biofoundries worldwide call for a more collaborative environment to share standard data formats and tools to cope with these problems and increase the biosafety of their protocols, especially when working with synthetic chromosomes. Firstly, data interchange formats, such as Antha (Synthace) and Autoprotocol (https://autoprotocol.org/, [94]), ensure that all critical information may be provided in a nondisruptive manner. Next, researchers have commonly focused on novel informatics for robustly designing and fabricating DNA devices to be collaboratively assembled into complex, large sequences (e.g., chromosomes) in multiple locations [95]. Such integrated cyberinfrastructure can help advance the biological understanding of the role of genetic components, regulatory mechanisms, and chromosome biophysics by improving our ability to manipulate large networks. Furthermore, open-source platforms, e.g., PyHamilton [96], can optimize pipetting in complex patterns for specific high-throughput experiments to remote-monitor hundreds of bacterial cultures in log-phase growth for days with no human intervention.

Among the collaborative initiatives around the world, first, the Global Biofoundries Alliance (https://biofoundries.org/) [97] is aimed at increasing the accessibility, efficiency, and reproducibility of construction pipelines worldwide by translating high-level descriptions of genetic circuits into requests for experiments performed by any member of the alliance around the globe. Then, the Bioindustrial Manufacturing and Design Ecosystem (https://biomade.org/) is developing a long-term, end-to-end bioindustrial manufacturing ecosystem to boost US competitiveness, derisk infrastructure investments, and expand the biomanufacturing education and workforce.

## 4. Engineering Multilayer Microfluidic Devices for Cell and Liquid Handling Operations

We consider “hardware” as the microfluidic components and their composed devices used for either assembling, screening, housing, or characterizing the Wetware so that together they meet the user-defined functional and performance specifications. Creating complex hardware devices, e.g., intelligent bioelectronic microfluidic solutions [20], requires exploring their components’ design space and performance characterization. Software tools and automated design workflows (Figure 2(b)) have helped researchers refine component geometries to tune the performance of composed devices (Figures 3 and 4). Next, the improvement of such tools has led microfluidic technologies to modernize the handling and flow of fluids in experiments where temporal-spatial control is required. The flow of reagents, cells, and chemicals through microfluidic features, such as microvalves, is controlled and peripheral instruments [15] (Figure 3). In addition, precise control over external variables, such as temperature [98] and acidic or oxidative stresses [99], also plays an essential role in creating environments for a precise characterization of biological systems. Ultimately, we expect such components, devices, and peripherals to facilitate the temporal description of serial biomolecular reactions in multistep biosynthesis processes [100].

Microfluidic components can be used to engineer complex microfluidic devices. Ideally, these composite microfluidic and genetic devices should be selected from their independent libraries of components based on their physical characteristics and roles, measured dynamic behavior and constraints, and ability to perform the user-desired function from a single abstract description. Then, software tools aid the extraction of experimental metrics, refinement, tuning parameters, and selected candidates. For example, to help in the design process and selection of optimal devices, machine learning techniques have been used to train models for finding the microfluidic droplet generator’s best geometries and operational parameters based on biological performance [56]. A similar approach could be applied to an entire library of components to enable system-level predictive microfluidic design.

Software tools should model and simulate the dynamics of a microfluidic device based on the performance and geometries of characterized components that compose them. Simultaneously, the design of microfluidic devices and the search for optimal performance output should consider the on-chip biological systems’ performance metrics [101]. The most common performance metrics that can be obtained and analyzed are, e.g., when the biological system presents quasi-steady-state dynamics. In that analysis, dynamical properties include, e.g., the fluorescent signal from a cell population, signal thresholds after changing in chemical (promoter-inducer) concentrations, and cell toxicity measured from optical density measurements. As described, electronics can be integrated into microfluidic chips to automate the collection of these read-outs.

In addition, microfabrication techniques promise to revolutionize how one can assess properties, rules, and constraints for constructing biological systems and testing their temporal and spatial performance using integrated microfluidic devices and microscopy [34]. A comprehensive review has identified key microfluidic design frameworks [78], which can help users physically design and fabricate devices with complex continuous flow-based microfluidic networks for housing and testing genetic systems [102]. These workflows consist mainly of software tools and instructions for designing and fabricating multilayer devices, varying in material required, fluid operation, biological system performance, and fabrication technology.

However, despite multiple studies and applications, droplet-based systems are limited to those based on biological systems’ steady-state dynamics. So, unlike keeping initial microenvironment variables as in droplets, systems such as continuous-flow, multilayer microfluidic devices enable temporal control of fluids and cells commonly require continuous monitoring and tracking of signals over time.

Microfluidic very-large-scale integration (mVLSI) devices can be used for high-throughput measurement of dynamic biological systems. Droplet-based platforms can be designed from micromechanical valve-based components, such as pumps, routers, and multiplexers (MUX), to feed cells with fresh media and other liquid combinations and remove excess cells and molecules. These routing components connect inputs and outputs with cell traps and independent units to grow and maintain cells for many generations (Figure 3).

To ensure modularity and simplicity of design and fabrication, we have implemented a hybrid fabrication approach of mVLSIs. First, multilayer microfluidic devices are composed of control and flow layers and an elastic membrane (e.g., silicon) (e.g., Figure 2(b)). The flow and control layers of the device can be fabricated from a polycarbonate substrate, then aligned and assembled using a pressure-activated adhesive (PSA) acrylic membrane to seal the device. Fluids flow through the flow layer in closed pathways/channels, while vacuum/air pressure is applied in the control layer to active/inactive microvalves, thus controlling the flow in the flow pathways of the flow layer [103]. The mechanism for opening and closing valves accounts for the deformation of the membrane layer through pressure and vacuum difference generated by a cavity on top of the layer, connected to pressure and control lines through channels and inlets located in the control layer.

Next, there are many ways cell traps can be designed as long-term microenvironments. Recent studies have shown different microfluidic layouts, e.g., linear trench [104], monolayer “daughter” device [105], and “mother” machine [106]. Using single-cell microscopy, they act as controllable microenvironments for studying programmable genetic circuits and their expected dynamical properties [107]. Such PDMS chips are fabricated using soft lithography [103]. They can trap bacterial cells due to monolayer chambers of around one *μ*m in height. They can be used to trap and maintain cells growing long-term (order of weeks) while having their performance assessed by phase-contrast and fluorescence time-lapse microscopy imaging. However, soft lithography has an expensive and slow design cycle: when cell traps are unnecessary and microfluidic geometries do not exceed 100-350 *μ*m, such as for fluidic routing, laser-cutting and micromilling are highly recommended to enable rapid fabrication.

Finally, coupling microfluidic devices with microscopy techniques promises to generate controlled experiments to test composed wetware and hardware. Image and data analysis workflows have been developed to measure and track the fluorescence intensity of single cells or an entire cell population for multiple cell generations over time. As required, peripheral, inverted microscopes, equipped with white brightfield or fluorescence brightfield, are used for, potentially, multichannel, multilocation, time-lapse imaging generation [89, 108]. In these experiments, external variables and experimental conditions can test and observe cells trapped in various external contexts. Thus, unlike most robotic technologies, microenvironments allow for a broader range of environmental conditions (i.e., from optimal to suboptimal) to which circuits can be exposed.

This approach creates a mechanism for fast assessing a priori biological systems’ multidimensional performance and potential application for deployable systems in real-life point-of-care applications [109, 110], for example, *in loco* heavy metal sensing [111] or synthetic compound produced by cellular consortia [19] embedded into deployable microenvironments based on either paper [109], thermoplastics [56], and soft lithography and PDMS [112].

## 5. Controlling Flow Operations in Multilayer Microfluidic Devices

mVLSI are devices based on assembling control and flow layers used to create component features, such as valves and pumps, to form complex fluidic functions, such as multiplexers and routers [69]. Defining the combination of valves and ports that should be active/inactive in control and flow paths determines the type of operation chips will perform. Such systems harness a complexity that benefits from design automation and control tools. For example, in Figure 5, in a multilayer microenvironment designed using 3DuF, the operation for loading cells requires two pathways in the multiplexers to be active while feeding two independent reservoirs simultaneously.

Tools for designing valves of mVLSI devices include Biostream [113], Micado [114], and others [18, 71] for placing and routing valves for a given flow layer. Next, resistive electric circuits can represent the hydraulic resistance of microfluidic networks to help optimize the number of valves in complex chips [79]. This work developed a CAD tool to draw fluidic operations and constraints and translate microfluidic layouts into an optimized resistance electrical profile, featuring adjustable resistors with constant hydraulic resistances. Then, it translates profiles into optimized hydraulic arrangements and resistive microfluidic networks suitable for fabrication. Technology-agnostic frameworks, such as “Fluigi” [73], promise to assist users lacking fabrication skills to design mVLSI devices irrespective of fabrication procedures downstream.

Tools for controlling valves for mVLSI include computer scripts that map valves to physical solenoids, allowing the switch between valves and pathways based on user-defined operational modes (Figure 5) [115]. Similarly, a recently developed tool (http://github.com/cidarlab/guide_tool.git) can generate fluidic operations from 3DuF designs. The example shown operates with up to seven independent operational modes (not all described here), such as (i) loading cells in each chemostat using a specific inlet of the multiplex, (ii) cleaning all main channels after cell loading, and (iii) cleaning of all main channels after reservoir filling. In addition, every operation requires a specific list of valves to be closed or open at the time. Finally, the occupancy of valves reveals interdependencies that help users design fewer valves and programmatically control their functioning.

Finally, mVLSI solutions from embedded systems can assist in exploring critical properties of biological systems and their relationship with multiple environment contexts. For example, custom spatial-temporally distributed chips can be designed to isolate different cells, keep stable coculture growth, and allow resource exchange. Microfluidic systems with interchangeable experimental conditions require tools for controlling peripherals, e.g., syringe pumps and thermostats, for flow control and microenvironment temperature maintenance. This strategy can potentially allow for many permutations and rapid biological interaction assessment, helping define the optimal microfluidic features per experimental condition. In the future, the study of the most relevant relationships between genetic and microenvironment contexts is expected to generate a microfluidic toolset of model-guided designs and deployable microfluidic devices for potential biological application.

## 6. Conclusion

This article offers a vision where hardware, software, and wetware can holistically become an end-to-end forward engineering framework for developing novel synthetic biological systems. In this framework, wetware and hardware creation will utilize common resources to ensure that the device can be created by anyone with access to the initial specification independent of their practical expertise. Ideally, a series of formalized design transformations and instructions for physical implementations and a library of characterized components will be connected through open-source databases that allow for the engineering of “wetware” (by cloud laboratories, e.g., DNA Biofoundries) and “hardware” (by microfluidic facilities). This would only be possible provided users commonly follow the same standards, formats, rules, and constraints for files and tools.

This work draws inspiration from embedded electronics, which are currently made with a “hardware, software, codesign” approach. This article provides insights into how software tools and off-the-shelf hardware can be applied to design and fabricate novel biological and microfluidic devices from abstract descriptions which can extract the functional, structural, and performance requirements of the design. It also presents the state-of-the-art of three topics, discussing their main technical challenges and technical development opportunities. If this approach becomes widely available, we believe it can contribute to developing context-independent biomaterials and embedded microfluidic devices for bioremediation, energy, and agriculture applications.

The key challenges that remain in this approach are as follows:

(1) What initial specification should be decomposed into the wetware, hardware, and software data structures? What is its syntax? What are its semantics? What users is it targeting? How does it remain application-agnostic while still being useful? This is a trade-off between expressiveness and constraining the design space
(2) How many wetware and hardware components are available, composable, and characterized? This approach relies heavily on its building blocks and how well the compositions can be modeled and physically assembled. How much does context matter?
(3) Integrating and reliably executing these complex systems across organizations and institutions. The techniques involved in related workflows will be key to being amendable to various laboratory conditions, experimental/computational expertise, and resources

It is becoming clear that in the 21^st^ century, biology holds the key to solving many of our most pressing challenges. It is highly unlikely that our current manual approaches will scale and effectively address these challenges. Therefore, it is even more urgent that we as a community start to consider the various elements needed to explore these solutions effectively. Wetware solutions alone are unlikely to work in isolation. It is our hope that by gaining inspiration from other fields, we will be able to decompose functions into the wetware, hardware, and software that together can meet these challenges and, in the process, make future challenges more easily addressable, results more replicable, and democratize the process for a wider variety of engineers and scientists.

## References

[1] D. Endy, “Foundations for engineering biology,” *Nature*, vol. 438, no. 7067, pp. 449–453, 2005 16306983 10.1038/nature04342

[2] A. S. Khalil, and J. J. Collins, “Synthetic biology: applications come of age,” *Nature Reviews. Genetics*, vol. 11, no. 5, pp. 367–379, 2010 10.1038/nrg2775 PMC2896386 20395970

[3] A. A. K. Nielsen, T. H. Segall-shapiro, and C. A. Voigt, “Advances in genetic circuit design: novel biochemistries, deep part mining, and precision gene expression,” *Current Opinion in Chemical Biology*, vol. 17, no. 6, pp. 878–892, 2013 24268307 10.1016/j.cbpa.2013.10.003

[4] M. A. Khatun, M. A. Hoque, Y. Zhang, L. Ting, L. Cui, N.-Y. Zhou, and Y. Feng, “Bacterial consortium-based sensing system for detecting organophosphorus pesticides,” *Analytical Chemistry*, vol. 90, no. 17, pp. 10577–10584, 2018 30060656 10.1021/acs.analchem.8b02709

[5] S. Atsumi, T. Hanai, and J. C. Liao, “Non-fermentative pathways for synthesis of branched-chain higher alcohols as biofuels,” *Nature*, vol. 451, no. 7174, pp. 86–89, 2008 18172501 10.1038/nature06450

[6] X. Jin, and I. H. Riedel-Kruse, “Biofilm lithography enables high-resolution cell patterning via optogenetic adhesin expression,” *Proceedings of the National Academy of Sciences*, vol. 115, no. 14, pp. 3698–3703, 2018 10.1073/pnas.1720676115 PMC5889658 29555779

[7] J. Dumont, D. Euwart, B. Mei, S. Estes, and R. Kshirsagar, “Human cell lines for biopharmaceutical manufacturing: history, status, and future perspectives,” *Critical Reviews in Biotechnology*, vol. 36, no. 6, pp. 1110–1122, 2016 26383226 10.3109/07388551.2015.1084266 PMC5152558

[8] J. J. Tabor, H. M. Salis, Z. B. Simpson, A. A. Chevalier, A. Levskaya, E. M. Marcotte, C. A. Voigt, and A. D. Ellington, “A synthetic genetic edge detection program,” *Cell*, vol. 137, no. 7, pp. 1272–1281, 2009 19563759 10.1016/j.cell.2009.04.048 PMC2775486

[9] S. R. Scott, M. Omar Din, P. Bittihn, L. Xiong, L. S. Tsimring, and J. Hasty, “A stabilized microbial ecosystem of self-limiting bacteria using synthetic quorum-regulated lysis,” *Nature Microbiology*, vol. 2, pp. 1–9, 2017 10.1038/nmicrobiol.2017.83 PMC5603288 28604679

[10] J. Beal, R. Weiss, D. Densmore, A. Adler, E. Appleton, J. Babb, S. Bhatia, N. Davidsohn, T. Haddock, J. Loyall, R. Schantz, V. Vasilev, and F. Yaman, “An end-to-end workflow for engineering of biological networks from high-level specifications,” *ACS Synth Biology*, vol. 1, no. 8, pp. 317–331, 2012 10.1021/sb300030d 23651286

[11] D. M. Densmore, and S. Bhatia, “Bio-design automation: software + biology + robots,” *Trends in Biotechnology*, vol. 32, no. 3, pp. 111–113, 2014 24269087 10.1016/j.tibtech.2013.10.005

[12] T. Jones, S. M. D. Oliveira, C. J. Myers, C. A. Voigt, and D. Densmore, “Genetic circuit design automation with Cello 2.0,” *Nature Protocols*, vol. 17, no. 4, pp. 1097–1113, 2022 35197606 10.1038/s41596-021-00675-2

[13] S.,. M. Abul, B. L. Hassan, N. Samper, L. Hang, C. A. Rushlow, G. Jiménez, S. Y. Shvartsman, and S. Sinha, “A systematic ensemble approach to thermodynamic modeling of gene expression from sequence data,” *Cell Systems*, vol. 1, no. 6, pp. 396–407, 2015 27136354 10.1016/j.cels.2015.12.002 PMC5094195

[14] J. A. McLaughlin, C. J. Myers, Z. Zundel, G. Mısırlı, M. Zhang, I. D. Ofiteru, A. Goñi-Moreno, and A. Wipat, “SynBioHub: a standards-enabled design repository for synthetic biology,” *ACS Synthetic Biology*, vol. 7, no. 2, pp. 682–688, 2018 29316788 10.1021/acssynbio.7b00403

[15] H. Huang, and D. Densmore, “Integration of microfluidics into the synthetic biology design flow,” *Lab on a Chip*, vol. 14, no. 18, pp. 3459–3474, 2014 25012162 10.1039/c4lc00509k

[16] J. R. Moffitt, J. B. Lee, and P. Cluzel, “The single-cell chemostat: an agarose-based, microfluidic device for high-throughput, single-cell studies of bacteria and bacterial communities,” *Lab on a Chip*, vol. 12, no. 8, pp. 1487–1494, 2012 22395180 10.1039/c2lc00009a PMC3646658

[17] Y. Khilko, P. D. Weyman, J. I. Glass, M. D. Adams, M. A. McNeil, and P. B. Griffin, “DNA assembly with error correction on a droplet digital microfluidics platform,” *BMC Biotechnology*, vol. 18, no. 1, p. 37, 2018 29859085 10.1186/s12896-018-0439-9 PMC5984785

[18] G. Liu, H. Huang, Z. Chen, H. Lin, H. Liu, X. Huang, and W. Guo, “Design automation for continuous-flow microfluidic biochips: a comprehensive review,” *Integration*, vol. 82, pp. 48–66, 2021

[19] C. E. Lawson, W. R. Harcombe, R. Hatzenpichler, S. R. Lindemann, F. E. Löffler, M. A. O’Malley, H. G. Martín, B. F. Pfleger, L. Raskin, O. S. Venturelli, and D. G. Weissbrodt, “Common principles and best practices for engineering microbiomes,” *Nature Reviews Microbiology*, vol. 17, no. 12, pp. 725–741, 2019 31548653 10.1038/s41579-019-0255-9 PMC8323346

[20] J. L. Terrell, T. Tschirhart, J. P. Jahnke, K. Stephens, Y. Liu, H. Dong, M. M. Hurley, M. Pozo, R. McKay, C. Y. Tsao, H.-C. Wu, G. Vora, G. F. Payne, D. N. Stratis-Cullum, and W. E. Bentley, “Bioelectronic control of a microbial community using surface-assembled electrogenetic cells to route signals,” *Nature Nanotechnology*, vol. 16, no. 6, pp. 688–697, 2021 10.1038/s41565-021-00878-4 33782589

[21] J. K. Jung, K. K. Alam, M. S. Verosloff, D. A. Capdevila, M. Desmau, P. R. Clauer, J. W. Lee, P. Q. Nguyen, P. A. Pastén, S. J. Matiasek, J.-F. Gaillard, D. P. Giedroc, J. J. Collins, and J. B. Lucks, “Cell-free biosensors for rapid detection of water contaminants,” *Nature Biotechnology*, vol. 38, no. 12, pp. 1451–1459, 2020 10.1038/s41587-020-0571-7 PMC7718425 32632301

[22] B. Alberts, A. Johnson, J. Lewis, M. Raff, K. Roberts, and P. Walter *Molecular Biology of the Cel l[Wilson and Tim Hunt]*, H. John, Ed., Garland Science, 4th ed., New York, USA, 2002

[23] D. M. Wolf, and A. P. Arkin, “Motifs, modules and games in bacteria,” *Current Opinion in Microbiology*, vol. 6, no. 2, pp. 125–134, 2003 12732301 10.1016/s1369-5274(03)00033-x

[24] J. Mäkelä, M. Kandhavelu, S. M. D. Oliveira, J. G. Chandraseelan, J. Lloyd-Price, J. Peltonen, O. Yli-Harja, and A. S. Ribeiro, “In vivo single-molecule kinetics of activation and subsequent activity of the arabinose promoter,” *Nucleic Acids Research*, vol. 41, no. 13, pp. 6544–6552, 2013 23644285 10.1093/nar/gkt350 PMC3711423

[25] J. P. Stratford, C. L. A. Edwards, M. J. Ghanshyam, D. Malyshev, M. A. Delise, Y. Hayashi, and M. Asally, “Electrically induced bacterial membrane-potential dynamics correspond to cellular proliferation capacity,” *Proceedings of the National Academy of Sciences of the United States of America*, vol. 116, no. 19, pp. 9552–9557, 2019 31000597 10.1073/pnas.1901788116 PMC6511025

[26] E. O. Puchkov, “Intercellular signaling in microbial world: a panoramic view,” *Biochemistry (Moscow) Supplement Series A: Membrane and Cell Biology*, vol. 10, no. 1, pp. 1–10, 2016

[27] L. J. Kahl, and D. Endy, “A survey of enabling technologies in synthetic biology,” *Journal of Biological Engineering*, vol. 7, no. 1, pp. 13–18, 2013 23663447 10.1186/1754-1611-7-13 PMC3684516

[28] A. S. Khalil, T. K. Lu, C. J. Bashor, C. L. Ramirez, N. C. Pyenson, J. Keith Joung, and J. J. Collins, “A synthetic biology framework for programming eukaryotic transcription functions,” *Cell*, vol. 150, no. 3, pp. 647–658, 2012 22863014 10.1016/j.cell.2012.05.045 PMC3653585

[29] Z. Kis, H. Sant’Ana Pereira, T. Homma, R. M. Pedrigi, and R. Krams, “Mammalian synthetic biology: emerging medical applications,” *Journal of Royal Society Interface*, vol. 12, no. 106, article 20141000, p. 18, 2015 10.1098/rsif.2014.1000 PMC4424663 25808341

[30] C. J. Paddon, P. J. Westfall, D. J. Pitera, K. Benjamin, K. Fisher, D. McPhee, M. D. Leavell, A. Tai, A. Main, D. Eng, D. R. Polichuk, K. H. Teoh, D. W. Reed, T. Treynor, J. Lenihan, H. Jiang, M. Fleck, S. Bajad, G. Dang, D. Dengrove, D. Diola, G. Dorin, K. W. Ellens, S. Fickes, J. Galazzo, S. P. Gaucher, T. Geistlinger, R. Henry, M. Hepp, T. Horning, T. Iqbal, L. Kizer, B. Lieu, D. Melis, N. Moss, R. Regentin, S. Secrest, H. Tsuruta, R. Vazquez, L. F. Westblade, L. Xu, M. Yu, Y. Zhang, L. Zhao, J. Lievense, P. S. Covello, J. D. Keasling, K. K. Reiling, N. S. Renninger, and J. D. Newman, “High-level semi-synthetic production of the potent antimalarial artemisinin,” *Nature*, vol. 496, no. 7446, pp. 528–532, 2013 23575629 10.1038/nature12051

[31] A. Mashaghi, and C. Dekker, “Systems and synthetic biology approaches to cell division,” *Systems and Synthetic Biology*, vol. 8, no. 3, pp. 173–178, 2014 25136378 10.1007/s11693-014-9132-z PMC4127179

[32] A. K. Nielsen, B. S. Der, J. Shin, P. Vaidyanathan, D. Densmore, and C. A. Voigt, “Genetic circuit design automation,” *Science*, vol. 352, no. 6281, pp. aac7341–aac7364, 2016 27034378 10.1126/science.aac7341

[33] L. B. Andrews, A. A. K. Nielsen, and C. A. Voigt, “Cellular checkpoint control using programmable sequential logic,” *Science*, vol. 361, no. 6408, 2018 10.1126/science.aap8987 30237327

[34] J. G. Chandraseelan, G. Jerome, S. M. D. Oliveira, A. Häkkinen, H. Tran, I. Potapov, A. Sala, M. Kandhavelu, and A. S. A. S. Ribeiro, “Effects of temperature on the dynamics of the LacI-TetR-CI repressilator,” *Molecular BioSystems*, vol. 9, no. 12, pp. 3117–3123, 2013 24104727 10.1039/c3mb70203k

[35] J. Hasty, D. McMillen, and J. J. Collins, “Engineered gene circuits,” *Nature*, vol. 420, no. 6912, pp. 224–230, 2002 12432407 10.1038/nature01257

[36] J. A. N. Brophy, and C. A. Voigt, “Principles of genetic circuit design,” *Nature Methods*, vol. 11, no. 5, pp. 508–520, 2014 24781324 10.1038/nmeth.2926 PMC4230274

[37] A. Lashkaripour, C. Rodriguez, L. Ortiz, and D. Densmore, “Performance tuning of microfluidic flow-focusing droplet generators,” *Lab on a Chip*, vol. 19, no. 6, pp. 1041–1053, 2019 30762047 10.1039/c8lc01253a

[38] O. S. Venturelli, M. Tei, S. Bauer, J. G. Leanne, C. J. Chan, C. J. Petzold, and A. P. Arkin, “Programming mRNA decay to modulate synthetic circuit resource allocation,” *Nature Communications*, vol. 8, no. 1, p. 15128, 2017 10.1038/ncomms15128 PMC5414051 28443619

[39] J. Kim, A. Darlington, M. Salvador, J. Utrilla, and J. I. Jiménez, “Trade-offs between gene expression, growth and phenotypic diversity in microbial populations,” *Current Opinion in Biotechnology*, vol. 62, pp. 29–37, 2020 31580950 10.1016/j.copbio.2019.08.004 PMC7208540

[40] T. Miyamoto, S. Razavi, R. DeRose, and T. Inoue, “Synthesizing biomolecule-based Boolean logic gates,” *ACS Synthic Biology*, vol. 2, no. 2, pp. 72–82, 2013 10.1021/sb3001112 PMC3603578 23526588

[41] M. A. Marchisio, and J. Stelling, “Computational design tools for synthetic biology,” *Current Opinion in Biotechnology*, vol. 20, no. 4, pp. 479–485, 2009 19758796 10.1016/j.copbio.2009.08.007

[42] D. Sprinzak, and M. B. Elowitz, “Reconstruction of genetic circuits,” *Nature*, vol. 438, no. 7067, pp. 443–448, 2005 16306982 10.1038/nature04335

[43] K. Keutzer, A. R. Newton, J. M. Rabaey, and A. Sangiovanni-Vincentelli, “System-level design: orthogonalization of concerns and platform-based design,” *IEEE Transactions on Computer-Aided Design of Integrated Circuits and Systems*, vol. 19, no. 12, pp. 1523–1543, 2000

[44] M. F. Soto, J. Agustin Rodriguez, R. Pablo, and P. R. Fillottrani, “System C/TLM flow for SoC design and verification,” in *In 2015 Argentine School of Micro-Nanoelectronics, Technology and Applications (EAMTA)*, Villa Maria, Argentina, 2015, pp. 37–42

[45] P. D. Mulani, “SoC level verification using System Verilog,” in *In 2009 Second International Conference on Emerging Trends in Engineering & Technology*, Nagpur, India, 2009, pp. 378–380

[46] K. Hampson, “Technical evaluation of the systems modeling language (SysML),” *Procedia Computer Science*, vol. 44, pp. 403–412, 2015

[47] N. E. Grandel, K. R. Gamas, and M. R. Bennett, “Control of synthetic microbial consortia in time, space, and composition,” *Trends in Microbiology*, vol. 29, no. 12, p. 1105, 2021 10.1016/j.tim.2021.04.001 33966922

[48] S. Hengoju, M. Tovar, D. D. K. W. Man, S. Buchheim, and M. A. Rosenbaum, “Droplet microfluidics for microbial biotechnology,” *Advances in Biochemical Engineering/Biotechnology*, Springer Berlin Heidelberg, Berlin, Heidelberg, 2020 10.1007/10_2020_140 32888037

[49] T. Thorsen, R. W. Roberts, F. H. Arnold, and S. R. Quake, “Dynamic pattern formation in a vesicle-generating microfluidic device,” *Physical Review Letters*, vol. 86, no. 18, pp. 4163–4166, 2001 11328121 10.1103/PhysRevLett.86.4163

[50] K. Ahn, J. Agresti, H. Chong, M. Marquez, and D. A. Weitz, “Electrocoalescence of drops synchronized by size-dependent flow in microfluidic channels,” *Applied Physics Letters*, vol. 88, no. 26, article 264105, 2006

[51] K. Ahn, C. Kerbage, T. P. Hunt, R. M. Westervelt, D. R. Link, and D. A. Weitz, “Dielectrophoretic manipulation of drops for high-speed microfluidic sorting devices,” *Applied Physics Letters*, vol. 88, no. 2, article 024104, 2006

[52] A. R. Abate, T. Hung, P. Mary, J. J. Agresti, and D. A. Weitz, “High-throughput injection with microfluidics using picoinjectors,” *Proceedings of the National Academy of Sciences*, vol. 107, no. 45, pp. 19163–19166, 2010 10.1073/pnas.1006888107 PMC2984161 20962271

[53] W.-w. Liu, and Y. Zhu, “"Development and application of analytical detection techniques for droplet- based microfluidics"-a review,” *Analytica Chimica Acta*, vol. 1113, pp. 66–84, 2020 32340670 10.1016/j.aca.2020.03.011

[54] C. Philip, I. K. Gach, K. Peter, N. J. Hilson, and A. K. Singh, “Droplet microfluidics for synthetic biology,” *Lab on a Chip*, vol. 17, no. 20, pp. 3388–3400, 2017 28820204 10.1039/c7lc00576h

[55] G. Taguchi, “Introduction to quality engineering: designing quality into products and processes,” vol. 658, no. 562, p. T3, 1986

[56] A. Lashkaripour, C. Rodriguez, N. Mehdipour, R. Mardian, D. McIntyre, L. Ortiz, J. Campbell, and D. Densmore, “Machine learning enables design automation of microfluidic flow-focusing droplet generation,” *Nature Communications*, vol. 12, no. 1, p. 25, 2021 10.1038/s41467-020-20284-z PMC7782806 33397940

[57] D. McIntyre, A. Lashkaripour, and D. Densmore, “Active learning for efficient microfluidic design automation,” *IWBDA 2020*, 2020

[58] M. O. Din, A. Martin, I. Razinkov, N. Csicsery, J. Hasty, J. Hasty, and J. Hasty, “Interfacing gene circuits with microelectronics through engineered population dynamics,” *Science Advances*, vol. 6, no. 21, pp. 1–8, 2020 10.1126/sciadv.aaz8344 PMC7244307 32494744

[59] B. Shannon, C. G. Zamora-Chimal, L. Postiglione, D. Salzano, C. S. Grierson, L. Marucci, N. J. Savery, and M. di Bernardo, “In vivofeedback control of an antithetic molecular-titration motif inEscherichia coliusing microfluidics,” *ACS Synthetic Biology*, vol. 9, no. 10, pp. 2617–2624, 2020 32966743 10.1021/acssynbio.0c00105

[60] A. R. Abate, J. J. Agresti, and D. A. Weitz, “Microfluidic sorting with high-speed single-layer membrane valves,” *Applied Physics Letters*, vol. 96, no. 20, article 203509, 2010

[61] J. W. Kotula, S. Jordan Kerns, L. A. Shaket, L. Siraj, J. J. Collins, J. C. Way, and P. A. Silver, “Programmable bacteria detect and record an environmental signal in the mammalian gut,” *Proceedings of the National Academy of Sciences*, vol. 111, no. 13, pp. 4838–4843, 2014 10.1073/pnas.1321321111 PMC3977281 24639514

[62] A. C. Siegel, S. S. Shevkoplyas, D. B. Weibel, D. A. Bruzewicz, A. W. Martinez, and G. M. Whitesides, “Cofabrication of electromagnets and microfluidic systems in poly(dimethylsiloxane),” *Angewandte Chemie*, vol. 118, no. 41, pp. 7031–7036, 2006 10.1002/anie.200602273 17001718

[63] J.-H. So, and M. D. Dickey, “Inherently aligned microfluidic electrodes composed of liquid metal,” *Lab on a Chip*, vol. 11, no. 5, pp. 905–911, 2011 21264405 10.1039/c0lc00501k

[64] A. Sciambi, and A. R. Abate, “Generating electric fields in PDMS microfluidic devices with salt water electrodes,” *Lab on a Chip*, vol. 14, no. 15, pp. 2605–2609, 2014 24671446 10.1039/c4lc00078a PMC4079735

[65] D. McIntyre, A. Lashkaripour, and D. Densmore, “Rapid and inexpensive microfluidic electrode integration with conductive ink,” *Lab on a Chip*, vol. 20, no. 20, pp. 3690–3695, 2020 32895672 10.1039/d0lc00763c

[66] R. N. Alnahhas, J. J. Winkle, A. J. Hirning, B. Karamched, W. Ott, K. Josić, and M. R. Bennett, “Spatiotemporal dynamics of synthetic microbial consortia in microfluidic devices,” *ACS Synthetic Biology*, vol. 8, no. 9, pp. 2051–2058, 2019 31361464 10.1021/acssynbio.9b00146 PMC6754295

[67] B. Said, and D. Or, “Synthetic microbial ecology: engineering habitats for modular consortia,” *Frontiers in Microbiology*, vol. 8, p. 1125, 2017 28670307 10.3389/fmicb.2017.01125 PMC5472676

[68] J. Vrana, O. de Lange, Y. Yang, G. Newman, A. Saleem, A. Miller, C. Cordray, S. Halabiya, M. Parks, E. Lopez, and S. Goldberg, “Aquarium: open-source laboratory software for design, execution and data management,” *Synthetic Biology*, vol. 6, no. 1, p. ysab006, 2021 34151028 10.1093/synbio/ysab006 PMC8209617

[69] J. McDaniel, B. Parker, and P. Brisk, “Simulated annealing-based placement for microfluidic large scale integration (mLSI) chips,” in *In 2014 22nd International Conference on Very Large Scale Integration (VLSI-SoC)*, Playa del Carmen, Mexico, 2014, pp. 1–6

[70] B. Crites, R. Sanka, J. Lippai, J. McDaniel, P. Brisk, and D. Densmore, “ParchMint: a microfluidics benchmark suite,” in *In 2018 IEEE International Symposium on Workload Characterization (IISWC)*, Raleigh, NC, 2018, pp. 78–79

[71] W. H. Minhass, J. McDaniel, M. Raagaard, P. Brisk, P. Pop, and J. Madsen, “Scheduling and fluid routing for flow-based microfluidic laboratories-on-a-chip,” *IEEE Transactions on Computer-Aided Design of Integrated Circuits and Systems*, vol. 37, no. 3, pp. 615–628, 2018

[72] I. E. Araci, and P. Brisk, “Recent developments in microfluidic large scale integration,” *Current Opinion in Biotechnology*, vol. 25, pp. 60–68, 2014 24484882 10.1016/j.copbio.2013.08.014

[73] H. Huang, and D. Densmore, “Fluigi,” *ACM Journal on Emerging Technologies in Computing Systems*, vol. 11, no. 3, pp. 1–19, 2014

[74] R. Sanka, B. Crites, J. McDaniel, P. Brisk, and D. Densmore, “Specification, integration, and benchmarking of continuous flow microfluidic devices: invited paper,” in *In 2019 IEEE/ACM International Conference on Computer-Aided Design (ICCAD)*, Westminster, CO, USA, 2019a, pp. 1–8

[75] B. G. Wong, C. P. Mancuso, S. Kiriakov, C. J. Bashor, and A. S. Khalil, “Precise, automated control of conditions for high-throughput growth of yeast and bacteria with eVOLVER,” *Nature Biotechnology*, vol. 36, no. 7, pp. 614–623, 2018 10.1038/nbt.4151 PMC6035058 29889214

[76] A. Lashkaripour, R. Silva, and D. Densmore, “Desktop micromilled microfluidics,” *Microfluidics and Nanofluidics*, vol. 22, no. 3, p. 31, 2018

[77] R. Sanka, J. Lippai, D. Samarasekera, S. Nemsick, and D. Densmore, “3D _*μ*_ F - interactive design environment for continuous flow microfluidic devices,” *Scientific Reports*, vol. 9, no. 1, p. 9166, 2019 31235804 10.1038/s41598-019-45623-z PMC6591506

[78] E. E. Tsur, “Computer-aided design of microfluidic circuits,” *Annual Review of Biomedical Engineering*, vol. 22, no. 1, pp. 285–307, 2020 10.1146/annurev-bioeng-082219-033358 32343907

[79] G. Linshiz, E. Jensen, N. Stawski, C. Bi, N. Elsbree, H. Jiao, J. Kim, R. Mathies, J. D. Keasling, and N. J. Hillson, “End-to-end automated microfluidic platform for synthetic biology: from design to functional analysis,” *Journal of Biological Engineering*, vol. 10, no. 1, p. 3, 2016 26839585 10.1186/s13036-016-0024-5 PMC4736182

[80] D. I. Walsh, D. S. Kong, S. K. Murthy, and P. A. Carr, “Enabling microfluidics: from clean rooms to makerspaces,” *Trends in Biotechnology*, vol. 35, no. 5, pp. 383–392, 2017 28162773 10.1016/j.tibtech.2017.01.001 PMC6812491

[81] A. Casini, M. Storch, G. S. Baldwin, and T. Ellis, “Bricks and blueprints: methods and standards for DNA assembly,” *Nature Reviews Molecular Cell Biology*, vol. 16, no. 9, pp. 568–576, 2015 26081612 10.1038/nrm4014

[82] T. Dinmukhamed, Z. Huang, Y. Liu, X. Lv, J. Li, D. Guocheng, and L. Liu, “Current advances in design and engineering strategies of industrial enzymes,” *Systems Microbiology and Biomanufacturing*, vol. 1, no. 1, pp. 15–23, 2021

[83] C. E. Sequeiros-Borja, B. Surpeta, and J. Brezovsky, “Recent advances in user-friendly computational tools to engineer protein function,” *Briefings in Bioinformatics*, vol. 22, no. 3, p. bbaa 150, 2021 10.1093/bib/bbaa150 PMC8138880 32743637

[84] I. Otero-Muras, and P. Carbonell, “Automated engineering of synthetic metabolic pathways for efficient biomanufacturing,” *Metabolic Engineering*, vol. 63, pp. 61–80, 2021 33316374 10.1016/j.ymben.2020.11.012

[85] R. Young, M. Haines, M. Storch, and P. S. Freemont, “Combinatorial metabolic pathway assembly approaches and toolkits for modular assembly,” *Metabolic Engineering*, vol. 63, pp. 81–101, 2021 33301873 10.1016/j.ymben.2020.12.001

[86] E. M. LeProust, B. J. Peck, K. Spirin, H. B. McCuen, B. Moore, E. Namsaraev, and M. H. Caruthers, “Synthesis of high-quality libraries of long (150mer) oligonucleotides by a novel depurination controlled process,” *Nucleic Acids Research*, vol. 38, no. 8, pp. 2522–2540, 2010 20308161 10.1093/nar/gkq163 PMC2860131

[87] M. Septak, “Kinetic studies on depurination and detritylation of CPG-bound intermediates during oligonucleotide synthesis,” *Nucleic Acids Research*, vol. 24, no. 15, pp. 3053–3058, 1996 8760893 10.1093/nar/24.15.3053 PMC146050

[88] R. Chen, N. J. Emery, M. Pavan, and S. M. D. Oliveira, “Laboratory protocol automation: a modular DNA assembly and bacterial transformation case study,” in *Proceedings of IWBDA2020*, 2020,

[89] S. M. D. Oliveira, J. G. Chandraseelan, A. Häkkinen, N. S. M. Goncalves, O. Yli-Harja, S. Startceva, and A. S. Ribeiro, “Single-cell kinetics of a repressilator when implemented in a single-copy plasmid,” *Molecular BioSystems*, vol. 11, no. 7, pp. 1939–1945, 2015 25923804 10.1039/c5mb00012b

[90] S. V. Iverson, T. L. Haddock, J. Beal, and D. M. Densmore, “CIDAR MoClo: improved MoClo assembly standard and newE. colipart library enable rapid combinatorial design for synthetic and traditional biology,” *ACS Synthetic Biology*, vol. 5, no. 1, pp. 99–103, 2016 26479688 10.1021/acssynbio.5b00124

[91] K. Clancy, and C. A. Voigt, “Programming cells: towards an automated ‘genetic compiler’,” *Current Opinion in Biotechnology*, vol. 21, no. 4, pp. 572–581, 2010 20702081 10.1016/j.copbio.2010.07.005 PMC2950163

[92] C. A. Voigt, “Genetic parts to program bacteria,” *Current Opinion in Biotechnology*, vol. 17, no. 5, pp. 548–557, 2006 16978856 10.1016/j.copbio.2006.09.001

[93] R. Weiss, S. Basu, S. Hooshangi, A. Kalmbach, D. Karig, R. Mehreja, and I. Netravali, “Genetic circuit building blocks for cellular computation, communications, and signal processing,” *Natural Computing*, vol. 2, no. 1, pp. 47–84, 2003

[94] M. Bates, A. J. Berliner, J. Lachoff, P. R. Jaschke, and E. S. Groban, “Wet lab accelerator: a web-based application democratizing laboratory automation for synthetic biology,” *ACS Synthetic Biology*, vol. 6, no. 1, pp. 167–171, 2017 27529358 10.1021/acssynbio.6b00108

[95] N. Ostrov, J. Beal, D. Tom Ellis, B. Gordon, B. J. Karas, H. H. Lee, S. C. Lenaghan, G. Stracquadanio, A. Trefzer, and J. S. Bader, “Technological challenges and milestones for writing genomes,” *Science*, vol. 366, no. 6463, pp. 310–312, 2019 31624201 10.1126/science.aay0339

[96] E. J. Chory, D. W. Gretton, E. A. DeBenedictis, and K. M. Esvelt, “Enabling high-throughput biology with flexible open-source automation,” *Molecular Systems Biology*, vol. 17, no. 3, p. e9942, 2021 33764680 10.15252/msb.20209942 PMC7993322

[97] N. Hillson, M. Caddick, Y. Cai, J. A. Carrasco, M. W. Chang, N. C. Curach, D. J. Bell, R. le Feuvre, D. C. Friedman, X. Fu, N. D. Gold, M. J. Herrgård, M. B. Holowko, J. R. Johnson, R. A. Johnson, J. D. Keasling, R. I. Kitney, A. Kondo, C. Liu, V. J. J. Martin, F. Menolascina, C. Ogino, N. J. Patron, M. Pavan, C. L. Poh, I. S. Pretorius, S. J. Rosser, N. S. Scrutton, M. Storch, H. Tekotte, E. Travnik, C. E. Vickers, W. S. Yew, Y. Yuan, H. Zhao, and P. S. Freemont, “Building a global alliance of biofoundries,” *Nature Communications*, vol. 10, no. 1, p. 2040, 2019 10.1038/s41467-019-10079-2 PMC6506534 31068573

[98] S. M. D. Oliveira, A. Häkkinen, J. Lloyd-Price, H. Tran, V. Kandavalli, and A. S. Ribeiro, “Temperature-dependent model of multi-step transcription initiation in Escherichia coli based on live single-cell measurements,” *PLoS Computational Biology*, vol. 12, no. 10, pp. 1–18, 2016 10.1371/journal.pcbi.1005174 PMC5085040 27792724

[99] A. Gupta, J. Lloyd-Price, S. M. D. Oliveira, O. Yli-Harja, A.-B. A.-B. Muthukrishnan, and A. S. Ribeiro, “Robustness of the division symmetry inEscherichia coliand functional consequences of symmetry breaking,” *Physical Biology*, vol. 11, no. 6, article 66005, 2014 10.1088/1478-3975/11/6/066005 25382420

[100] G. W. Roell, J. Zha, R. R. Carr, M. A. Koffas, S. S. Fong, and Y. J. Tang, “Engineering microbial consortia by division of labor,” *Microbial Cell Factories*, vol. 18, no. 1, p. 35, 2019 30736778 10.1186/s12934-019-1083-3 PMC6368712

[101] M. R. Bennett, and J. Hasty, “Microfluidic devices for measuring gene network dynamics in single cells,” *Nature Reviews. Genetics*, vol. 10, no. 9, pp. 628–638, 2009 10.1038/nrg2625 PMC2931582 19668248

[102] J. Stricker, S. Cookson, M. R. Bennett, W. H. Mather, L. S. Tsimring, and J. Hasty, “A fast, robust and tunable synthetic gene oscillator,” *Nature*, vol. 456, no. 7221, pp. 516–519, 2008 18971928 10.1038/nature07389 PMC6791529

[103] M. A. Unger, H.-P. Chou, T. Thorsen, A. Scherer, and S. R. Quake, “Monolithic microfabricated valves and pumps by multilayer soft lithography,” *Science*, vol. 288, no. 5463, pp. 113–116, 2000 10753110 10.1126/science.288.5463.113

[104] N. Q. Balaban, J. Merrin, R. Chait, L. Kowalik, and S. Leibler, “Bacterial persistence as a phenotypic switch,” *Science*, vol. 305, no. 5690, pp. 1622–1625, 2004 15308767 10.1126/science.1099390

[105] O. Mondragón-Palomino, T. Danino, J. Selimkhanov, L. Tsimring, and J. Hasty, “Entrainment of a population of synthetic genetic oscillators,” *Science*, vol. 333, no. 6047, pp. 1315–1319, 2011 21885786 10.1126/science.1205369 PMC4841678

[106] P. Wang, L. Robert, J. Pelletier, W. L. Dang, F. Taddei, A. Wright, and S. Jun, “Robust growth of _*Escherichia coli*_,” *Current Biology*, vol. 20, no. 12, pp. 1099–1103, 2010 20537537 10.1016/j.cub.2010.04.045 PMC2902570

[107] A. Miano, M. J. Liao, and J. Hasty, “Inducible cell-to-cell signaling for tunable dynamics in microbial communities,” *Nature Communications*, vol. 11, no. 1, 2020 10.1038/s41467-020-15056-8 PMC7055273 32132536

[108] S. M. D. Oliveira, R. Neeli-Venkata, N. S. M. Goncalves, J. A. Santinha, L. Martins, H. Tran, J. Mäkelä, A. Gupta, M. Barandas, A. Häkkinen, J. Lloyd-Price, J. M. Fonseca, and A. S. Ribeiro, “Increased cytoplasm viscosity hampers aggregate polar segregation in Escherichia coli,” *Molecular Microbiology*, vol. 99, no. 4, pp. 686–699, 2016 26507787 10.1111/mmi.13257

[109] K. Pardee, A. A. Green, D. Tom Ferrante, E. Cameron, A. Daleykeyser, P. Yin, and J. J. Collins, “Paper-based synthetic gene networks,” *Cell*, vol. 159, no. 4, pp. 940–954, 2014 25417167 10.1016/j.cell.2014.10.004 PMC4243060

[110] S. Slomovic, K. Pardee, and J. J. Collins, “Synthetic biology devices for in vitro and in vivo diagnostics,” *Proceedings of the National Academy of Sciences of the United States of America*, vol. 112, no. 47, pp. 14429–14435, 2015 26598662 10.1073/pnas.1508521112 PMC4664311

[111] X. Wan, F. Volpetti, E. Petrova, C. French, S. J. Maerkl, and B. Wang, “Cascaded amplifying circuits enable ultrasensitive cellular sensors for toxic metals,” *Nature Chemical Biology*, vol. 15, no. 5, pp. 540–548, 2019 30911179 10.1038/s41589-019-0244-3

[112] L. Lin, M. Jie, F. Chen, J. Zhang, Z. He, and J.-M. Lin, “Efficient cell capture in an agarose–PDMS hybrid chip for shaped 2D culture under temozolomide stimulation,” *RSC Advances*, vol. 6, no. 79, pp. 75215–75222, 2016

[113] J. P. Urbanski, W. Thies, C. Rhodes, S. Amarasinghe, and T. Thorsen, “Digital microfluidics using soft lithography,” *Lab on a Chip*, vol. 6, no. 1, pp. 96–104, 2006 16372075 10.1039/b510127a

[114] N. Amin, W. Thies, and S. Amarasinghe, “Computer-aided design for microfluidic chips based on multilayer soft lithography,” in *In 2009 IEEE International Conference on Computer Design*, Lake Tahoe, CA, USA, 2009, pp. 2–9

[115] C. Watson, and S. Senyo, “All-in-one automated microfluidics control system,” *HardwareX*, vol. 5, article e00063, 2019 10.1016/j.ohx.2019.e00063 PMC6561480 31192312

